# Remembering a Virtual Museum Tour: Viewing Time, Memory Reactivation, and Memory Distortion

**DOI:** 10.3389/fpsyg.2022.869336

**Published:** 2022-04-14

**Authors:** Sarah Daviddi, Serena Mastroberardino, Peggy L. St. Jacques, Daniel L. Schacter, Valerio Santangelo

**Affiliations:** ^1^Department of Philosophy, Social Sciences & Education, University of Perugia, Perugia, Italy; ^2^Department of Psychology, Sapienza University of Rome, Rome, Italy; ^3^Department of Psychology, University of Alberta, Edmonton, AB, Canada; ^4^Department of Psychology, Harvard University, Cambridge, MA, United States; ^5^Functional Neuroimaging Laboratory, IRCCS Santa Lucia, Rome, Italy

**Keywords:** autobiographical memory, virtual tour, memory encoding, memory reactivation, memory recognition, false memories

## Abstract

A variety of evidence demonstrates that memory is a reconstructive process prone to errors and distortions. However, the complex relationship between memory encoding, strength of memory reactivation, and the likelihood of reporting true or false memories has yet to be ascertained. We address this issue in a setting that mimics a real-life experience: We asked participants to take a virtual museum tour in which they freely explored artworks included in the exhibit, while we measured the participants’ spontaneous viewing time of each explored artwork. In a following memory reactivation phase, participants were presented again with explored artworks (reactivated targets), followed by novel artworks not belonging to the same exhibit (activated lures). For each of these objects, participants provided a reliving rating that indexed the strength of memory reactivation. In the final memory recognition phase, participants underwent an old/new memory task, involving reactivated vs. baseline (i.e., non-reactivated) targets, and activated and baseline lures. The results showed that those targets that were spontaneously viewed for a longer amount of time were more frequently correctly recognized. This pattern was particularly true for reactivated targets associated with greater memory strength (a higher reliving rating). Paradoxically, however, lures that were presented after targets associated with higher reliving ratings in the reactivation phase were more often erroneously recognized as artworks encountered during the tour. This latter finding indicates that memory intrusions, irrespective of the viewing time, are more likely to take place and be incorporated into true memories when the strength of target memory is higher.

## Introduction

It is well established that memory is a reconstructive process (Schacter, 2001; Loftus, 2005), as supported by a number of studies showing that memory recall is prone to errors and distortions (see, for a review, Schacter et al., 2011). Memory reactivation mechanisms have been shown to play a key role in memory consolidation and memory recall: After an experience is encoded into memory, the spontaneous or instructed reactivation of that experience is thought to contribute to long-term memory stabilization and transformation of that trace, mostly by hippocampal–cortical connectivity (Tambini and Davachi, 2019). Memory reactivation can therefore be considered as a process that favors the opportunity for memory traces to be enhanced and updated with new information (Johnson and Chalfonte, 1994). However, memory reactivation also makes memories susceptible to physiological and behavioral interference (Scully et al., 2017). The dual effect of memory reactivation is well exemplified in the context of autobiographical memory by a “museum paradigm” (St. Jacques et al., 2013, 2015; St. Jacques and Schacter, 2013) that used a memory reactivation procedure to measure memory distortions. The participants were asked to take part in a self-guided audio tour of a museum wearing a body camera that automatically took photos every 15 s (encoding phase). After the tour, a memory reactivation phase took place, in which participants were presented with pictures taken from their tour (reactivated targets). Each reactivated target was immediately followed by the presentation of a novel lure photo from an alternative tour version (reactivation plus new information). Results from the subsequent recognition memory phase showed better performance for reactivated vs. non-reactivated targets, but also increased false alarms for lures, which were incorrectly recognized as items encountered during the tour.

These findings highlight that the reactivation procedure can selectively enhance—but also distort—autobiographical memories. However, it is currently unexplored whether reactivation mechanisms depend (and to what extent) on encoding-related features, such as the amount of time spent to view a specific item. In most studies of real-world memory, it is difficult to precisely control the amount of time that people spend viewing events. People may spontaneously spend more time viewing/interacting with stimuli that they find more interesting, and these differences in encoding time are known to improve memory (Li et al., 2020). Here we used virtual reality (VR) to examine how people’s natural viewing time with ecological valid experiences impacts their memories. A growing body of studies used VR to investigate episodic-autobiographical memory (Smith, 2019). Recent findings showed that VR experiences promote better autobiographical retrieval accuracy than conventional laboratory events (Kisker et al., 2021). To date, however, how learning mechanisms operate in VR and whether these processes are comparable to those operating in real life was scarcely investigated (Smith and Mulligan, 2021). Here we contributed to this issue by assessing memory of a virtual museum tour (VMT). This procedure allowed us to measure the spontaneous viewing time of each artwork explored in a setting that mimics real experience, that is, during the VMT. Two hours after, half of the artworks explored during the VMT were presented again to the participants (reactivated targets) and rated on reliving (Rubin et al., 2003). Each reactivated target was followed by novel information consisting of an artwork not belonging to the same exhibit (activated lures). We expected that a longer viewing time would promote higher reliving ratings, indicating greater strength of memory representation.

The day after the reactivation phase, the participants were administered an old/new recognition task, involving the presentation of reactivated/non-reactivated targets, and activated/non-activated lures (i.e., items that were previously presented in the reactivation phase or not, respectively). In agreement with the previous literature, we expected higher recognition accuracy for reactivated vs. non-reactivated targets, but also increased false alarms for activated vs. non-activated lures (St. Jacques et al., 2013). Moreover, we investigated the relationship between viewing time, strength of memory reactivation, and memory distortions (activated lures recognized as items encountered during the tour). If longer viewing time and/or greater memory strength of reactivated targets acts as a protective factor against memory distortions, we should find that correctly rejected lures are associated with those targets with longer viewing time and/or higher reliving rating. Conversely, increased target viewing time and/or reliving rating associated with increased false alarms (i.e., memory distortions) would paradoxically indicate that memory intrusions are more likely to be incorporated into true memories when the target viewing time and/or memory strength is higher.

## Materials and Methods

### Participants

Thirty-one healthy volunteers participated in this study, which was approved by the independent Ethics Committee of the IRCCS Santa Lucia and conducted in adherence to the tenets of the Declaration of Helsinki. Participants gave written informed consent to the study. Four of them were excluded from the analysis because of technical problems related to the video recording of the VMT, while another additional participant was excluded because he reported having misunderstood the experimental instructions at the end of the task, leaving 26 participants for the analysis (8 males; mean age: 29 years; SD: 9.44; range: 22–54 years). The final sample size was estimated on the basis of the previous literature using the same paradigm (St. Jacques et al., 2013) and on the basis of a power analysis conducted with G*Power 3.1.9.2 that confirmed statistical reliability with a sample size of 26 subjects (statistical power = 80%; medium effect size = 0.5; significance level = 0.05). None of the participants had already visited—in real life or virtually—the museum galleries that were explored during the experiment.

### Stimuli

**Figure 1:**
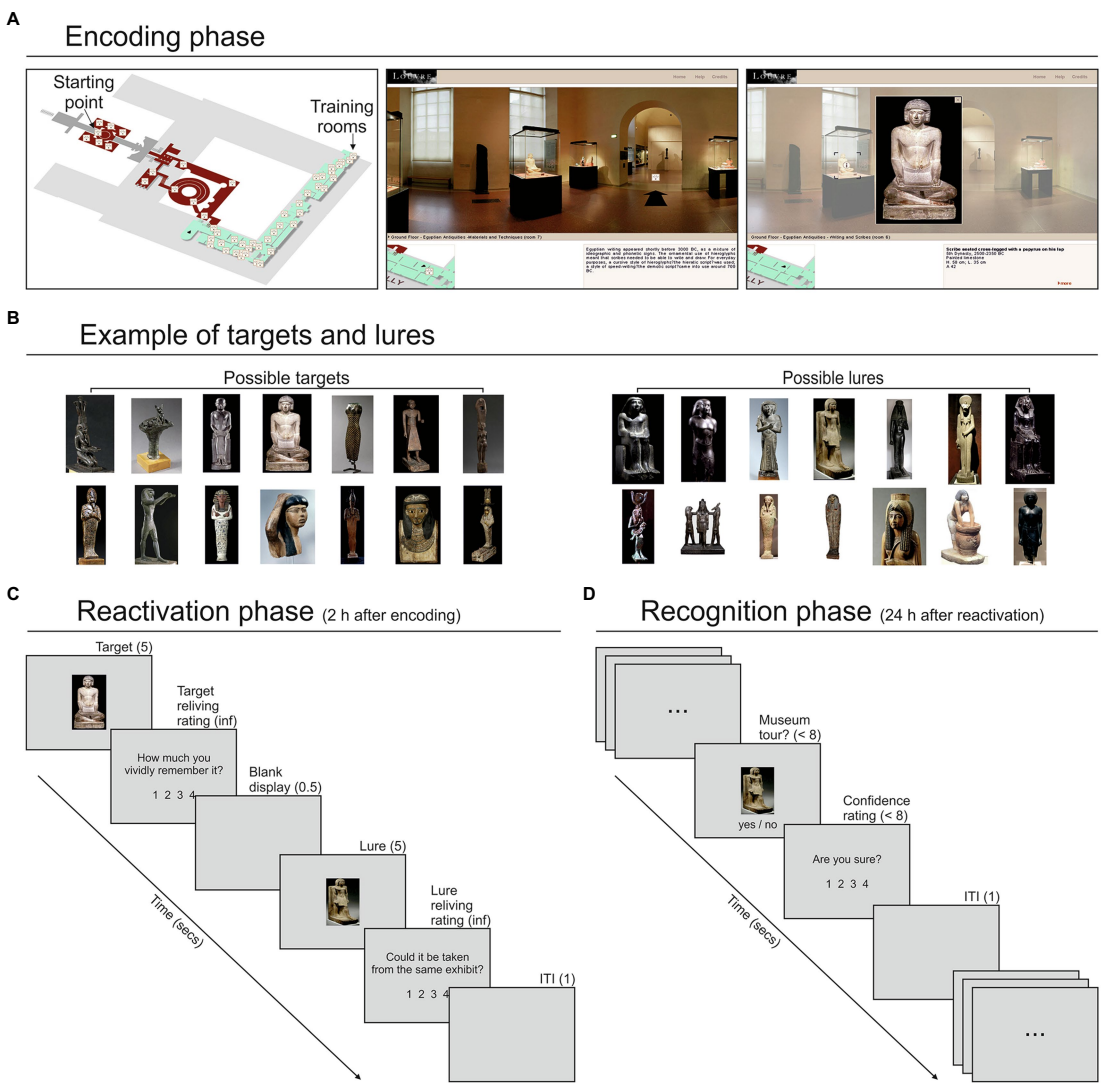
Virtual tour display and artworks examples. **(A)** Left panel: map of the explorable rooms of the Egyptian (in green) and Historic (in red) galleries of the Louvre Museum; middle panel: example of the view during the tour, including the display of a museum room, the position of the room in the map (bottom-left corner) and a brief description of the room (bottom-right corner); right panel: example of the close-up view of a clickable artwork—revealed by a circled “i” symbol (i.e., “information”) when moving the mouse—selected by the participant. **(B)** Targets and lures related to an example category, that is, “statuettes.” **(C)** Example of a trial of the memory reactivation phase that started 2 h after the end of the encoding phase. Each trial started with the presentation of a target object (i.e., an artwork explored in the encoding phase) for 5 s, and a following reliving rating; then, after a blank display of 0.5 s, a lure object, belonging to the same category of the target, was presented for 5 s, followed by a relatedness rating. **(D)** Example of a trial of the memory recognition phase that started 24 h after the reactivation phase. Each trial consisted in the presentation of an artwork. The participants judged whether this artwork was encountered during the virtual tour or not and then provided a confidence judgment. For each response, they had a maximum time of 8 s. ITI, inter-trial interval.

The stimuli consisted of 195 artworks placed in the Egyptian and Historical galleries of the Louvre Museum, explorable through a free and easily accessible virtual tour.^1^ The participants explored the galleries through a desktop VR system, that is, a virtual environment that uses a standard computer monitor and the keyboard/mouse as tools for interaction (Ausburn and Ausburn, 2004). This user-friendly approach drastically reduced for most people the time required to familiarize with the tool. The tour encompassed 23 rooms (Figure 1A; see Supplementary Methods). Each room could be explored by holding the mouse button and then moving it toward the left or right side of the screen. This procedure allowed a 360° exploration of each room. When the mouse pointer was in proximity of a door or a staircase, a black arrow appeared on the floor, allowing—with a mouse click—participants to move into a different part of the room or to an adjacent room. Alternatively, participants could move from one room to another by clicking on the corresponding location of the map, always available at the bottom-left corner of the display. During rooms/galleries exploration, participants encountered several artworks. Some of these artworks were clickable for a more detailed exploration through a close-up view and a related written description. These items were considered as target objects for the subsequent memory task. Targets were subdivided into several categories, each including a variable number of items (see Supplementary Methods). For each category, we found on the Internet an equal number of artworks (not exposed in the Louvre Museum) that served as lures in the reactivation/recognition phases (Figure 1B).

### Procedure

The procedure was modeled after (St. Jacques et al., 2013) and consisted of three phases:

#### Encoding

The encoding phase involved virtual exploration of the galleries previously described. Before starting the VMT, the participants were allowed 5 min of exploration—limited to two rooms of the Egyptian section that included 15 clickable artworks—to familiarize themselves with the navigation tools. No other rooms were explored during the training session, and the artworks explored during training were not used in the following phases. The main VMT started from the same predefined point for each participant. The participants were instructed to navigate across the rooms and search for clickable artworks. They were free to observe the artworks for as long as they wanted. For each participant, the exploration continued until 120 out of 195 clickable artworks were found and explored by clicking on them. A researcher (SD) positioned behind the participants observed the monitor and counted the number of explored artworks. For each participant, the entire VMT was video-recorded using Camstudio 2.7.2 for offline identification of the artworks individually explored, which allowed us to select the appropriate lures for the following memory reactivation phase. Lures were randomly extracted from the set of images associated with a given target category (e.g., if a participant explored 4 “statuettes” in the VMT, four lures were randomly selected from the related set and used in the following phases; Figure 1B).

#### Reactivation

After a 2-h delay from the end of the encoding phase (in which the participants were free to remain in or to leave the laboratory), the memory reactivation phase started (Figure 1C). This consisted of a computer task implemented with Cogent 1.30, running in Matlab 7.1 (duration about 15 min). Each trial started with the presentation of a random target (i.e., an artwork selected and explored by that participant during the VMT) for 5 s, followed by the question: “How much do you vividly remember it?”. Participants were instructed to retrieve their memory in as much detail as possible and then to rate their subjective sense of recollection or re-experience on a four-point scale from low to high, with no time limits. After the presentation of a blank display for 0.5 s, a lure item randomly selected from the same category of the previous target item was presented for 5 s, and participants were asked to make a perceptual decision, operationalized by the question: “Could it be taken from the same exhibit?”, answering again on a four-point scale from low to high, with no time limits. When a given lure was used in one trial it could not be used in a different trial for that participant. After an inter-trial interval (ITI) of 1 s, the following trial began. This task included 60 trials, involving 120 stimuli, 60 targets, and 60 lures.

#### Recognition

The recognition phase started approximately 24 h (24 h and 9 min ± 30 min) after the conclusion of the reactivation phase (Figure 1D) and consisted of a memory recognition task (again implemented with Cogent/Matlab). On each trial, the participants were presented with an artwork and their task was to judge, within 8 s, whether this item was encountered during the VMT (encoding phase). Then, they provided a confidence rating about their previous response (“Are you sure?” display) on a scale from 1 (“not at all”) to 4 (“completely”), within 8 s. After an ITI of 1 s, the next trial was presented. The recognition phase included 240 stimuli: 60 targets explored during the VMT and then reactivated in the reactivation phase (“reactivated targets”); 60 targets explored during the VMT but not presented in the reactivation phase (“baseline targets”); 60 lures presented during the reactivation phase (“activated lures”); 60 lures never presented to the participants (“baseline lures”). The stimuli were presented in a random order across two blocks of 120 stimuli each. Each block lasted about 12.5 min. Participants were allowed to rest a few minutes between the blocks.

## Results

**Figure 2:**
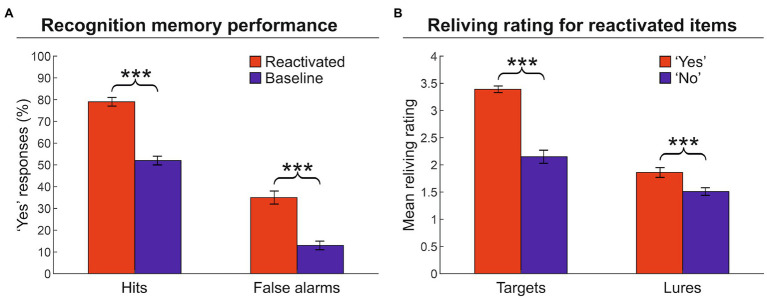
Memory recognition performance and reliving ratings. **(A)** Proportion of “yes” responses (± the standard error of the mean) for reactivated and baseline targets. The graph shows that the reactivation procedure increased both hits and false alarms. **(B)** Mean reliving ratings (± the standard error of the mean) for reactivated items expressed during the memory reactivation phase. The graph shows that higher reliving ratings are associated with an increased probability to recognized the artwork (target or lure) as an item encountered during the museum tour (i.e., a “yes” response). ^***^p < 0.001.

First, we computed the proportion of “yes” responses during the recognition memory phase, that is, the responses in which the participants indicated that the artwork was encountered during the VMT (hit targets). Participants remembered reactivated targets (79%) more frequently than baseline targets (52%) [hits, two-tailed paired *t*-test: *t*(25) = 15.4, *p* < 0.001, Cohen’s *d* = 3.190], indicating that the reactivation phase increased the number of hits (Figure 2A; see also Supplementary Table 1). However, the reactivation phase also increased the number of lures erroneously recognized as items encountered during the VMT, as evidenced by the significant difference between activated lures (35%) vs. baseline lures (13%) [false alarms: *t*(25) = 10.4, *p* < 0.001, *d* = 2.113]. Second, we examined the relationship between the degree of reliving during the reactivation phase and subsequent recognition memory performance, for reactivated items (both targets and lures) that were recognized as encountered (“yes” responses) or not (“no” responses) in the VMT. Hit targets were associated with greater reliving ratings as compared to not recognized targets [3.4 vs. 2.2; *t*(25) = 11.1, *p* < 0.001, *d* = 2.108] (Figure 2B). Similarly, erroneously recognized lures were associated with greater reliving ratings than correctly rejected lures [1.9 vs. 1.5; *t*(25) = 6.3, *p* < 0.001, *d* = 1.448]. These findings indicate that the quality/strength of reactivation affected the level of true and false memories: The greater the reliving rate, the higher the probability of recognizing that item as encountered during the VMT (a “yes” response at the memory recognition phase).

**Figure 3:**
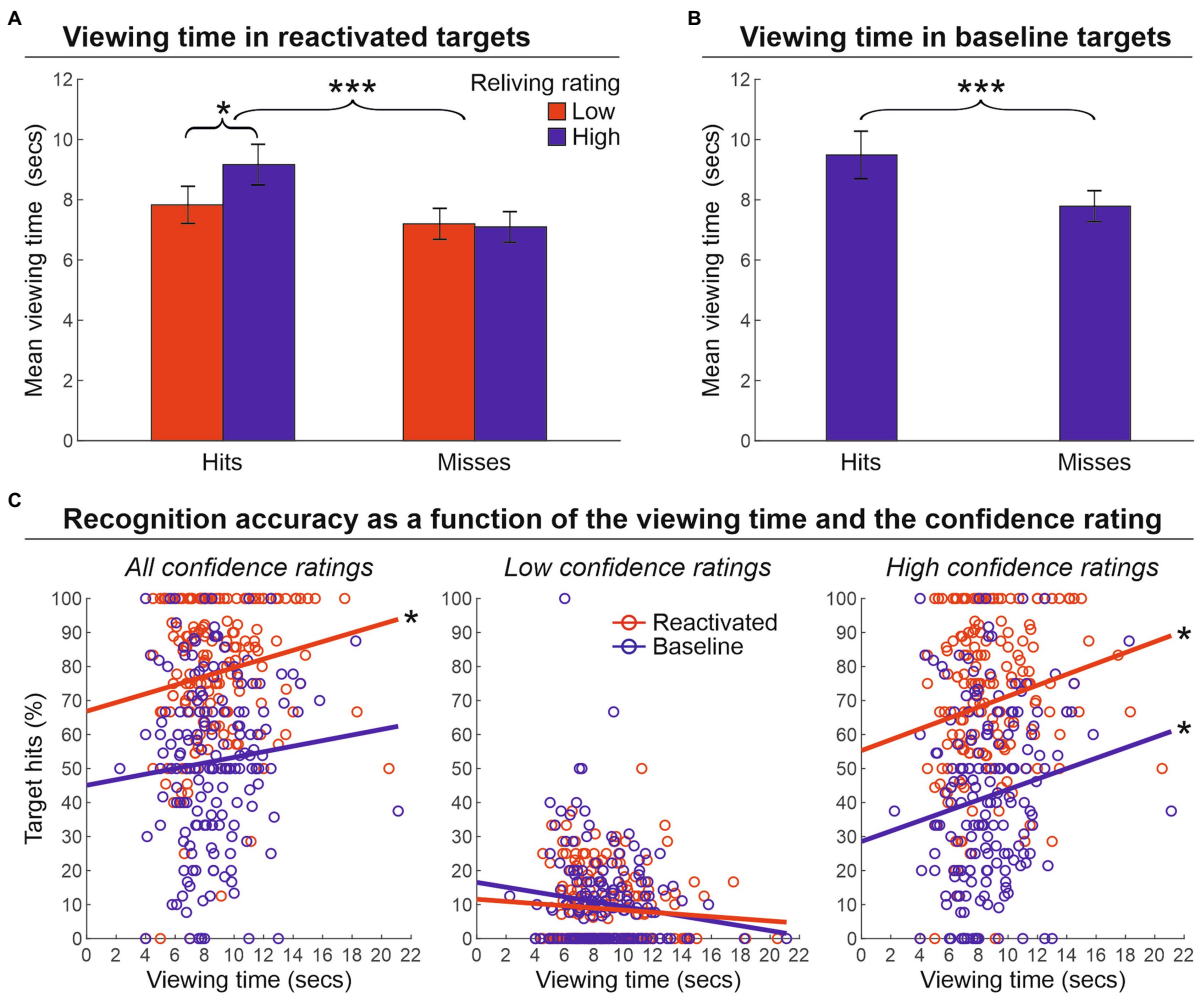
Viewing time. **(A)** Mean viewing time (± the standard error of the mean) in reactivated targets that were recognized (hits) or not (misses) at the memory recognition task (phase 3) according to the reliving rating reported during the reactivation procedure (phase 2). This shows greater viewing time for hits vs. misses, and also greater viewing time for high reliving rate hit targets. **(B)** Mean viewing time (± the standard error of the mean) in baseline targets, indicating a greater viewing time spent for non-reactivated targets subsequently recognized (hit) vs. missed in the recognition memory task (phase 3). **(C)** The relationship between recognition accuracy and viewing time. The graphs show that when higher confidence scores are considered, the recognition accuracy of both reactivated and baseline targets benefited from a longer viewing time. ^***^p < 0.001; ^*^p < 0.05.

Next, we evaluated the impact of the viewing time on the strength of memory reactivation (phase 2) for targets that were correctly recognized (hits) or not (missed; phase 3; see Supplementary Results for preliminary analyses of participants’ behavior at encoding). We conducted a two-way repeated-measure ANOVA with the factors of Performance outcome of reactivated targets (hits or misses) and Reliving rating (low, 1–2 scores, vs. high, 3–4 scores) on the viewing time (Figure 3A). This analysis revealed a main effect of the target performance outcome [*F*(1.25) = 15.4, *p* < 0.001, *ɳ*^2^ = 0.382], indicating that hit targets were encoded for a longer amount of time than missed targets (8.5 and 7.1 s, respectively). The main effect of reliving rating was not significant [*F*(1.25) = 2.5, *p* = 0.123, *ɳ*^2^ = 0.092]. Importantly, however, the reliving rating interacted with the target performance outcome [*F*(1.25) = 5.9, *p* = 0.023, *ɳ*^2^ = 0.190], indicating a longer viewing time for those targets that received a higher reliving rating (vs. low reliving rating), but only for hits (9.2 vs. 7.8 s) and not for missed targets (7.2 vs. 7.1 s). Increased viewing time was also found for hits (8.9 s) vs. missed (7.2 s) baseline targets [*t*(25) = 3.7, *p* < 0.001, *d* = 0.694] (Figure 3B). Furthermore, the relationship between the viewing time and target memory performance was also confirmed by an item-specific correlation analysis that crossed the average viewing time with the average recognition accuracy (percentage of hits) for each target object across participants. As illustrated in Figure 3C, we found a significant correlation between the viewing time and the percentage of hits for reactivated targets (*r* = 0.169, *p* = 0.023, *r*^2^ = 0.029), but not for baseline targets (*r* = 0.085, *p* = 0.257, *r*^2^ = 0.007; left panel). However, when considering high confidence ratings only (i.e., 3 and 4 scores; right panel), we found a significant correlation for both reactivated (*r* = 0.188, *p* = 0.011, *r*^2^ = 0.035) and baseline targets (*r* = 0.153, *p* = 0.041, *r*^2^ = 0.023), confirming that the recognition accuracy of both reactivated and baseline targets benefited from longer viewing time. Conversely, when considering low confidence ratings only (1 and 2 scores; central panel), no relation between recognition accuracy and viewing time was found, neither for reactivated (*r* = −0.083, *p* = 0.266, *r*^2^ = 0.007), nor for baseline targets (*r* = −0.135, *p* = 0.073, *r*^2^ = 0.018).

**Figure 4:**
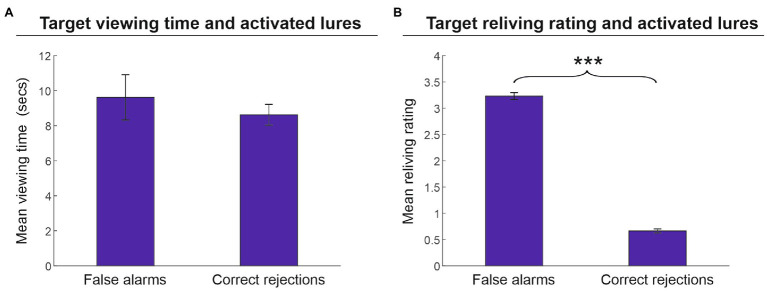
Memory recognition performance for activated lures. Mean viewing time **(A)** and mean reliving rating **(B)** (± the standard error of the mean) in reactivated targets associated with activated lures (phase 2) that were erroneously (false alarms) or correctly recognized (correct rejections) in phase 3. Target-related viewing time did not significantly affect lure recognition outcome **(A)**; by contrast, the target reliving rating was significantly higher in those lures that were erroneously recognized as artworks encountered in the tour compared to lures correctly rejected **(B)**. ^***^p < 0.001.

Finally, we assessed whether encoding-related parameters (i.e., viewing time and reliving ratings) affected the fate of lures in terms of false alarms and correct rejections. As illustrated in Figure 4A, we found that the performance outcome of the activated lures in the memory reactivation phase (i.e., false alarms vs. correct rejections) was not affected by the viewing time of the related targets (9.6 vs. 8.6 s, respectively) [*t*(25) = 1.1, *p* = 0.281, *d* = 0.216]. Conversely, the strength of target memory, as indexed by target reliving ratings, significantly affected the fate of lures (Figure 4B). Lures erroneously recognized as artworks encountered during the tour (false alarms) were associated with targets that received a higher reliving rate (3.2) as compared to lures that were correctly rejected (0.7) [*t*(25) = 4.6, *p* < 0.001, *d* = 9.918].

## Discussion

In terms of recognition memory performance, the results were highly consistent with the previous literature (St. Jacques and Schacter, 2013). This finding is interesting because, in contrast to a previous study (Diamond et al., 2020), it indicates that the VMT produces effects similar to the actual experience of a museum tour. Specifically, the reactivation phase produced an increased number of reactivated (vs. baseline) targets correctly recognized as artworks explored during the VMT, possibly acting as a retrieval practice phase (McDermott, 2021). However, the reactivation phase also produced an increased number of false alarms (i.e., artworks erroneously recognized as objects encountered in the tour) for activated vs. baseline lures. This effect might be interpreted as a failure of source monitoring (Johnson et al., 1993; Mitchell and Johnson, 2009), related to the capability of attributing a given artwork to the VMT or to information seen later in the reactivation phase. Alternatively, as suggested by St. Jacques and Schacter (2013), the increase of false alarms might be interpreted on the basis of those models that have linked memory reactivation with updating of existing memories (Johnson and Chalfonte, 1994; Hardt et al., 2010; Sederberg et al., 2011), as memory reactivation also involves the incorporation of new information into existing memories (Hupbach et al., 2007, 2009). The current design does not allow us to disentangle whether the increased number of false alarms was a consequence of memory updating or source confusion. However, our findings are consistent with the notion that memory reactivation—and, in general, memory retrieval—is an active process that can modify existing memories (Schacter, 2012; Scully et al., 2017).

We also found that the likelihood of recognizing an artwork as encountered during the tour was related to the reliving rating, both for reactivated targets and activated lures. This finding indicates that the strength of the (re)activation had a significant impact both on true and false memories (St. Jacques et al., 2013). The present study provides novel evidence about the generalizability of these reactivation-related effects, which were observed here using VR. Moreover, in the current study, we drastically reduced the time intervals among the three phases, from 48 (as in St. Jacques and Schacter, 2013) to 2 h. Also considering the well-known relationship between sleep and memory consolidation (Klinzing et al., 2019), a 48-h delay—involving two nights of sleep—typically allows memory consolidation into stable long-term memories (Lisman and Morris, 2001; Yang et al., 2009). Conversely, the 2-h delay used in the current experiment allowed us to investigate the impact of memory reactivation on much more recently encoded/less consolidated memories. Notwithstanding these temporal differences, we found similar findings compared to the previous studies (St. Jacques et al., 2013, 2015; St. Jacques and Schacter, 2013). This extends the previous literature, showing that memory reactivation enhances both target recognition and false alarms also at shorter delays.

The current findings also revealed a clear link between viewing time, strength of memory reactivation, and recognition accuracy. Correctly recognized targets were encoded for a longer amount of time than missed targets. This pattern was particularly evident for those reactivated targets associated with a higher reliving rating. This effect was also evident when switching to an object-based approach, that is, considering the fate of each single artwork in the tour. The longer a given artwork was encoded (averaging the viewing time across the participants) the higher the probability for that artwork to be correctly recognized, especially when the participants were more confident about the accuracy of their memory. Interestingly, while low confidence ratings are thought to be associated with familiarity-based recognition memory decisions, high confidence ratings are associated with recollection (Yonelinas, 2002; Evans and Wilding, 2012). The impact of viewing time therefore appears to be specifically linked to recollection-based memory judgments.

A number of studies have reported encoding-dependent benefits at retrieval in short-term memory tasks, suggesting that the likelihood to correctly retrieve information at short-term intervals is a function of the attentional resources devoted to that information at encoding (e.g., Fine and Minnery, 2009; Botta et al., 2010; Huebner and Gegenfurtner, 2011; Melcher and Piazza, 2011; Santangelo and Macaluso, 2013; Pedale and Santangelo, 2015; Santangelo et al., 2015; for reviews, see Gazzaley and Nobre, 2012; Santangelo, 2015). A similar link has been documented in long-term memory, showing for instance that the probability of successful retrieval is a function of the number of repetitions of the to-be-remembered material studied at encoding (Koutstaal et al., 1998). Consistently, we showed higher recognition accuracy as a function of spontaneous viewing time, especially for reactivated targets associated with higher reliving ratings. An increased viewing time therefore appears to promote successful recognition, possibly by creating stronger memory representation (Diekelmann et al., 2011). This suggestion is in good agreement with the non-monotonic plasticity hypothesis (Sinclair and Barense, 2019), according to which there is a U-shaped relationship between the strength of the memory reactivation and the degree of memory change. Weakly reactivated memories would not become labile, avoiding to influence synaptic plasticity. Moderate reactivation would instead induce optimal synaptic weakening, eliciting neural plasticity; and strong reactivation would strengthen synaptic connections, making memories resistant to later distortions and preserving accurate recall (Newman and Norman, 2010). Consistently, we showed that targets encoded for a longer time and then rated with higher reliving are more correctly retrieved than targets associated with lower reliving ratings.

One may argue that the viewing time and the related increase in the strength of memory reactivation may also act as a protective factor against memory distortions with respect to lure recognition. For instance, Marche and Howe (1995) reported that the susceptibility to misinformation decreased when the original information was encoded multiple times vs. once. The current findings did not reveal any effect of the viewing time on memory distortions. However, we found that lures were more likely erroneously recognized as artworks explored during the tour when they were presented after targets associated with higher reliving ratings in the reactivation phase. This finding suggests that new information (activated lures) was incorporated more easily into old information (reactivated targets) reactivated with greater memory strength. Incidentally, the specific question used during lure activation (“Could it be taken from the same exhibit?”) might have enhanced this effect by promoting imagination inflation (Garry et al., 1996). This finding can be interpreted in the light of the temporal context model (Sederberg et al., 2011), according to which memory reactivation reinstates the context from the original encoding occurred when the memory traces started to be formed. The reactivation of stronger memories associated with higher reliving ratings could lead to a greater reinstatement of the VMT (also in agreement with the non-monotonic plasticity hypothesis), leading participants to encode the category-matched lures within the same context of the target objects encountered during the tour. The reactivation of those targets associated with greater memory strength might have therefore favored the incorporation of false information (lures) to true information (the reactivated target associated with higher reliving ratings).

Some limitations should be also considered when interpreting the present findings. While the VMT allowed us to accurately measure the spontaneous viewing time of single artworks, there are several obvious differences from real-world experiences. For instance, in the real-world people would probably spend much longer time at each exhibit, and the social interaction among exhibit visitors might also have a deep impact on memory formation. Moreover, in the current study, we did not investigate several individual factors (curiosity and/or personal interest for specific objects/artworks, etc.) that might have contributed to driving some of the reported effects. Notwithstanding these important aspects that should be considered by future studies, we showed in a setting that mimics real experience that those artworks that were spontaneously viewed for a longer amount of time were more frequently correctly recognized as objects seen during the virtual tour. By contrast, the current findings did not reveal any effect of the viewing time on the proportion of memory distortions. We found instead that the proportion of memory distortions increased when novel artworks were associated with those artworks seen in the tour that elicited in the participants a stronger memory reactivation, irrespective of the viewing time. Overall, these findings suggest that longer viewing time promotes better memory recognition, but fails to act as a protective factor against memory distortions.

## Data Availability Statement

The raw data supporting the conclusions of this article will be made available by the authors, without undue reservation.

## Ethics Statement

The studies involving human participants were reviewed and approved by the Ethics Committee of the IRCCS Santa Lucia Foundation. The patients/participants provided their written informed consent to participate in this study.

## Author Contributions

SD collected the data. SD and VS analyzed and interpreted the data and wrote the first draft of the manuscript. All authors contributed, conceived, and designed the study to improve the quality of the manuscript with significant feedback.

## Funding

This work was supported by the Bial Foundation (Grant No. 75/20, to VS) and by the Italian Ministry of Health (RF-2019-12369567, to VS).

## Conflict of Interest

The authors declare that the research was conducted in the absence of any commercial or financial relationships that could be construed as a potential conflict of interest.

## Publisher’s Note

All claims expressed in this article are solely those of the authors and do not necessarily represent those of their affiliated organizations, or those of the publisher, the editors and the reviewers. Any product that may be evaluated in this article, or claim that may be made by its manufacturer, is not guaranteed or endorsed by the publisher.

## Footnotes

^1^ http://musee.louvre.fr/visite-louvre/index.html?defaultView=entresol.s489.p01&lang=ENG
