# Supplementary material for: Remembering a Virtual Museum Tour: Viewing Time, Memory Reactivation, and Memory Distortion
Source: Front Psychol. 2022 Apr 14;13:869336. doi: 10.3389/fpsyg.2022.869336 (PMC9048676; doi:10.3389/fpsyg.2022.869336)
Supplement: Supplementary file 1 [file Data_Sheet_1.pdf]

Supplementary Material

1 Supplementary Methods

The tour encompassed 23 rooms (19 Egyptian; 4 Historical). Within each room, the participant was provided with the name and the number of the room [e.g., "Egyptian Antiquities – Materials and Techniques (room 7)"], as well as a brief description related to the theme of the room (e.g., Egyptian writing), and the position of the room in the map (Fig. 1A, middle panel).

During the virtual museum tour, there were 195 explorable (clickable) artworks (an average of 8 artworks by each room) that could be located by moving the mouse on the scene, which revealed on these objects a circled "i" symbol (i.e., "information"). The explorable artworks that we used as targets were subdivided into 17 different categories: statues (6 items), paintings (27 items), half-busts (6 items), war accessories/weapons (4 items), structural elements (10 items), bas-reliefs (11 items), ivory objects (4 items), sphinxes (3 items), walls/steles (17 items), sarcophagi/mummies (13 items), papyri (5 items), showcases (7 items), jewels (8 items), statuettes (14 items), rock sculptures (6 items), wovens (2 items), various objects (37 items).

2 Supplementary Results

For completeness, we reported the participants' accuracy in the recognition task for reactivated and baseline targets (i.e., the percentage of "yes" responses) and for activated and baseline lures (i.e., the percentage of "no" responses) (Supplementary Table 1).

Targets		Lures	
Reactivated	Baseline	Activated	Baseline
79 ± 9	52 ± 12	65 ± 14	87 ± 10

Supplementary Table 1. Participants' accuracy (mean percentage ± standard deviation) in the recognition task for reactivated and baseline targets (i.e., "yes" responses) and for activated and baseline lures (i.e., "no" responses).

Before assessing the impact of the viewing time on participants' performance, we conducted several preliminary analyses to evaluate participants' behaviour at encoding. The viewing time for every single artwork explored during the VMT was computed by reviewing the video recorded for each participant's exploration. Overall, the mean time of exploration was 47.0 ± 9.5 min across participants, with a mean number of 6 ± 5 artworks selected and explored per room. On average, each artwork was explored for 8.8 ± 2.2 seconds by the participants, although the individual viewing time (as well as the reliving rating and recognition accuracy) of each artwork varied consistently across participants.

Then, we assessed whether there was consistency in the viewing time of the explored objects across participants by computing inter-subject correlation (Supplementary Figure 1A). For this analysis, we

calculated pairwise correlation (Pearson's r) among the viewing time of common explored artworks considering each possible permutation of the group of subjects. Overall, the correlation coefficients tended to be positive, ranging between -0.2 and 0.5. However, after Bonferroni's correction for multiple comparisons, only a few pairs of subjects showed consistency in the viewing time of the explored artworks. Similarly, no inter-subject correlations were found when considering the reliving rating of the explored targets (Supplementary Figure 1B), and only a few significant correlations in terms of the recognition accuracy of the explored targets were observed (Supplementary Figure 1C), meaning that both the reliving rating and the recognition accuracy for a given target varied consistently among participants. Overall, these analyses suggest a high level of individual differences in the viewing time, the reliving rating, and the related recognition outcome (i.e., hit or missed target) for the artworks explored in the tour.

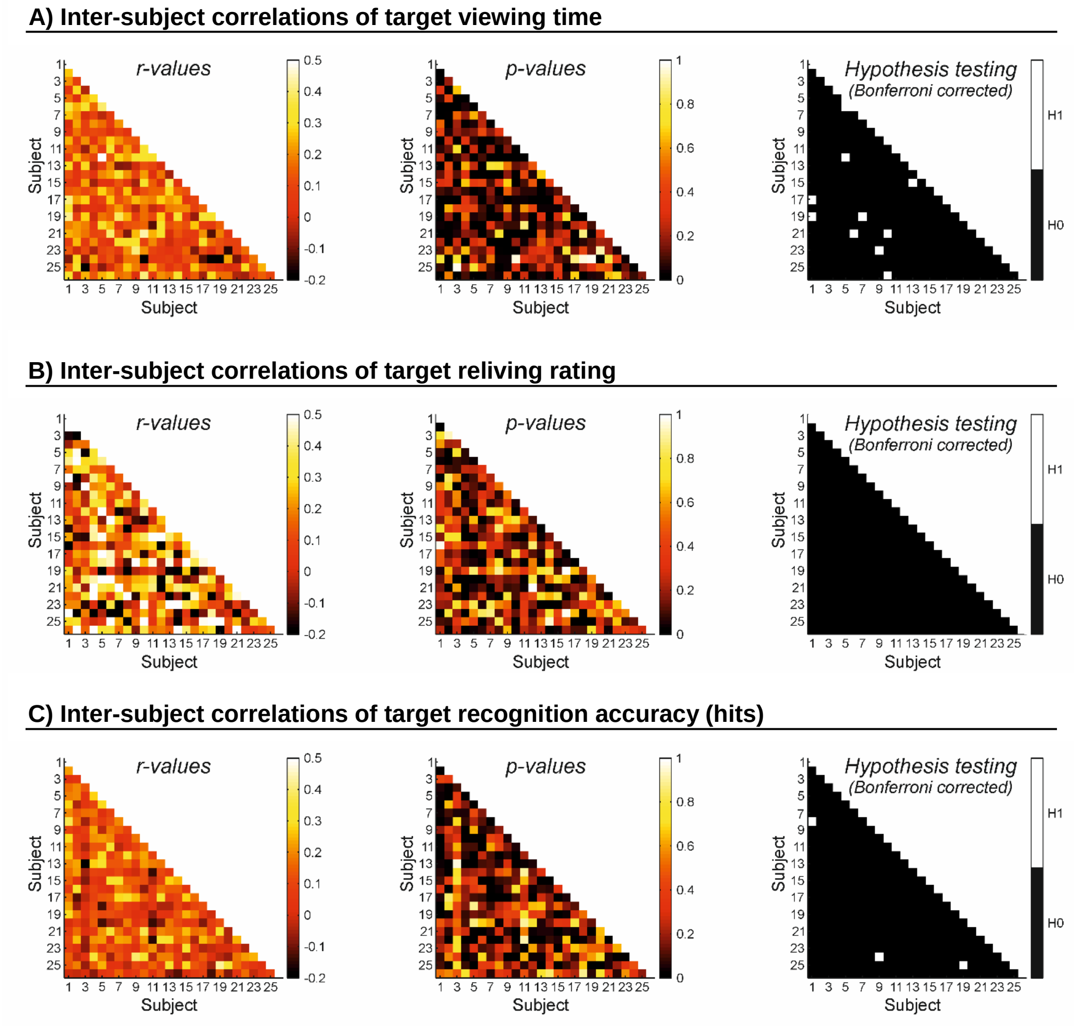

Supplementary Figure 1. Inter-subject correlations. Pairwise Pearson's r correlation (and related p-values) among the viewing time (A), the reliving rating (B), and the recognition accuracy (C) of common explored artworks considering each possible permutation of the group of subjects. After Bonferroni's correction, only a few pairs of subjects showed consistency in the viewing time and recognition accuracy of the explored artworks (A & C, right panels). No pair of subjects showed correlated target reliving rating (B, right panel).
